# Investigation of pyrethroid resistance mutations in *Linognathus stenopsis* lice collected from goats in western and northwestern Iran

**DOI:** 10.3389/fvets.2024.1380328

**Published:** 2024-06-14

**Authors:** Khadijeh Seydi-Gazafi, Mousa Tavassoli, Karim Mardani

**Affiliations:** ^1^Department of Pathobiology, Faculty of Veterinary Medicine, Urmia University, Urmia, Iran; ^2^Department of Food Hygiene and Quality Control, Faculty of Veterinary Medicine, Urmia University, Urmia, Iran

**Keywords:** *Linognathus stenopsis*, goat, pyrethroid resistance, Iran, VGSC gene

## Abstract

**Introduction:**

*Linognathus stenopsis* lice are an extensive parasitic concern in goat populations worldwide, posing significant economic and health risks. This study examined the identification of alleles of resistance to pyrethroid and mutations in *L. stenopsis* samples obtained from goats in five provinces in western and northwestern Iran.

**Methods:**

Morphological and molecular techniques were employed to identify the louse species. Molecular identification methods and gene sequencing were used to identify resistance-associated mutations in the voltage-gated sodium channel (VGSC) gene.

**Results and discussion:**

The results revealed that six amino acid substitutions, including threonine-to-isoleucine (T917I), leucine-to-phenylalanine (L920F), isoleucine-to-phenylalanine (I927F), phenylalanine-to-alanine (F928A), valine-to-arginine (V929R), and arginine-to-leucine (R930L) mutations, were present in the VGSC gene of *L. stenopsis* lice from various regions of Iran. These findings suggest the potential for pyrethroid resistance development in this louse species, highlighting the importance of integrated pest management (IPM) strategies. Such strategies, which combine selective insecticides, regular grooming, and environmental sanitation, are crucial for effectively managing *L. stenopsis* infestations and preserving the efficacy of pyrethroids for pest control. Moreover, the emergence of novel kdr mutations underscores the need for ongoing research into the molecular mechanisms underlying these mutations. This research is vital for developing strategies to combat pyrethroid resistance and maintaining the efficacy of insecticides in controlling lice.

## Introduction

*Linognathus stenopsis* is a type of blood-sucking louse that specifically infests goats. As an obligatory permanent ectoparasite, it causes considerable harm to hosts. Lice infestation presents a significant challenge for small ruminants, resulting in mortality, reduced productivity, fertility problems, and a decrease in the value of the skin for the tanning industry (1). Lice infestation in goats leads to hypersensitivity to proteins found in the saliva. This hypersensitivity causes the main clinical signs, such as irritation and hair loss. Excessive scratching can lead to secondary infections. Infestation can have significant effects on production and reproductive indices, resulting in anemia and abortion. Furthermore, weight loss and reproductive disorders contribute to economic loss in goat farming (2).

The occurrence of ectoparasites in sheep and goat flocks has been reported in Iran, but quantitative data are limited. These regions were selected for this study because of their specific geographical features and the likelihood of ectoparasite infestation. In addition, sheep and goat farming is a major animal husbandry practice in western and northwestern Iran (3).

One of the most important treatments for caprine pediculosis is the localized and external application of pyrethroids. Pesticide resistance has increased, resulting in partial inactivity of any product and/or increasingly massive dosage requirements. However, the indiscriminate use of insecticides can harm human and animal health, increasing the risk of chemical residuals in animals and animal-derived products (4). Pyrethroids are a class of highly selective insecticides that have emerged as valuable tools for controlling a diverse range of pest insects in public health, animal, and agricultural domains (5). Pyrethroids disrupt the normal activity of voltage-gated sodium channels in insects and trigger uncontrolled action potential bursts that rapidly induce exhaustion, paralysis, and death. Sodium channels undergo a series of transitions between an open, activated state and an inactivated, closed state. These transitions are critical for the generation and propagation of electrical signals (i.e., action potentials). Pyrethroids interfere with these transitions by preventing the inactivation and deactivation of sodium channels, leading to prolonged opening (6). VGSC gene generates and propagates action potentials in the most excitable cells. Pyrethroid insecticides are the primary targets of action because of their crucial role in regulating membrane excitability. One major mechanism of resistance is knockdown resistance (kdr), which is a natural defense mechanism developed by insects to combat the effects of insecticides, particularly pyrethroids. This resistance arises from the VGSC gene. When the VGSC gene are mutated, they become less responsive to pyrethroids, thereby rendering them less susceptible to toxic effects. Kdr has been documented in almost all medically and agriculturally important arthropod pest species. This widespread resistance has been primarily attributed to the excessive use of pyrethroids for several decades. Sustained exposure to pyrethroids has allowed insects to evolve and develop mutations in the VGSC gene, enabling them to withstand the effects of insecticides. The identification of specific mutations in lice that confer resistance to pyrethroids provides reliable molecular markers for rapidly assessing the prevalence of resistance alleles in field populations. Early detection of pyrethroid resistance is critical for implementing effective management strategies and delaying the further development of resistance. Consequently, developing a rapid and accurate resistance monitoring system using molecular markers is essential to prolong the efficacy of available insecticides (6, 7). The present study serves as a crucial first step toward evaluating resistance alleles in *L. stenopsis*, lice species collected from goats in five Iranian provinces.

## Materials and methods

### Description of study areas

The study was conducted from September 2018 to March 2019, encompassing five provinces in western and northwestern Iran: Lorestan, Hamadan, Kurdistan, West Azerbaijan, and Kermanshah. All the research sites were situated in the west and northwest of Iran. The climate of northwestern Iran is characterized by a harsh, snowy winter, temperate spring and autumn, and blazing, and dry summer. The western region experiences a humid continental climate, with colder winters and warmer summers. Sufficient precipitation and fertile vegetation underpin livestock farming in both regions, with cattle, sheep, goats, and horses being the primary domestic animals. In these regions pyrethroids are widely used. It was prompted by observations and unpublished reports from colleagues, which indicated that goat lice showed resistance to treatment.

### Study animals and sampling method

The requisite minimum sample size was determined using the formula described by Thrusfield (8). The sample size was calculated to achieve a 95% confidence level with a 5% absolute precision and an expected prevalence of 10%. As a result, 1,572 blood-sucking and chewing lice were directly removed from the bodies of the 420 goats in 20 farms of five provinces. A wide range of goats was used for the study, including males and females of various age groups, body conditions, agroecology, species, and flock types kept under an extensive management system.

### Ectoparasites collection

The coat-brushing technique was employed to collect lice from the skin of the host. The Lice was collected, preserved in individually labeled universal vials filled with 70% ethanol, and transported to the Parasitology Laboratory of the Faculty of Veterinary Medicine, Urmia University, Iran.

### Morphological identification

The coat-brushing technique was used to collect lice from the skin of the hosts. Samples were collected, preserved in individually labeled universal vials filled with 70% ethanol, and transported to the Parasitology Laboratory of the Faculty of Veterinary Medicine, Urmia University, Iran.

### Molecular identification

#### DNA extraction

Lice were individually homogenized in 100 mL of lysis buffer encased in liquid nitrogen. Following the addition of 10 mL of ribotinase, the mixture was incubated at 55°C for 1–2 h. The remaining steps of the DNA extraction protocol were performed meticulously according to the manufacturer’s instructions. This meticulous extraction method was employed to unequivocally identify the *L. stenopsis* species.

A 700-base pair (bp) segment of the mitochondrial cox1 gene was specifically amplified from DNA extracted from lice specimens using a standard polymerase chain reaction (PCR) protocol. The PCR reaction employed the primer pair COX1, consisting of 5′-GGCAACAAATCATAAAGATATTG G-3′ and 3′-GAA GGG TCA AAG AAT GAT GT-5′, to target the desired region for amplification (9). The PCR amplification was performed using a standardized PCR kit (Cinagen Company, Tehran) in a final reaction volume of 50 μL. The reaction mixture consisted of 5 μL of DNA extract, 5 μL of 10x PCR buffer, 4 μL of MgCl2 (50 mM), 1 μL of dNTP mix (100 μM), 1 μL of each primer (20 μM), and 1 μL of Taq polymerase. A negative control was included in each reaction to verify that the reaction components, except the DNA sample, functioned properly. PCR was performed using a Quants Biotech thermocycler (England, QB-96). The amplified PCR products were separated by gel electrophoresis on 1.5% agarose gel. After electrophoresis, the gel was visualized and photographed using a UV transilluminator (BTS-20 M; Japan).

### Molecular detection of specific allele

A 600-base pair (bp) segment of the VGSC gene was specifically amplified from DNA extracted from lice specimens using a standardized PCR protocol. PCR was performed using a pair of sense and antisense primers, designated VGSC-forward (5′-CTG GGCA ATC GCG ACT CTCT CCT CCC GCT G-3) and VGSC-reverse (5′- CTG CTGG CAG CCC CCG CCC G-3), to specifically target the desired region for amplification (10), Standardized PCR was performed using a Cinagen Company PCR kit (Tehran) in a final reaction volume of 50 μL. The reaction mixture consisted of 5 μL DNA extract, 5 μL 10x PCR buffer, 4 μL MgCl2 (50 mM), 1 μL dNTP mix (100 μM), 1 μL each primer (20 μM), and 1 μL Taq polymerase. A negative control was included in each reaction to verify the proper functioning of all reaction components apart from the DNA sample. Polymerase chain reaction (PCR) was performed using a Quants Biotech thermocycler (England, QB-96). The amplified PCR products were separated by gel electrophoresis on 1.5% agarose gel. Following electrophoresis, the gel was visualized and photographed using a UV transilluminator (BTS-20 M; Japan).

Standardized PCR was performed using validated positive controls and a non-template reaction mixture as a negative control. The extraction and negative controls were PCR-negative, ensuring the integrity of the PCR procedure. Amplified PCR products were purified using the MBST-IRAN kit. Eleven samples were sent to a takapouzist (Tehran, Iran) for DNA sequencing. Initial sequencing analysis was performed using Chromas 2.2.1. Sequencing results were registered online in NCBI’s Basic Local Alignment Search Tool (BLAST), along with the corresponding host and geographical information, to obtain unique accession numbers. Subsequently, data analysis was conducted by submitting the sequences to NCBI BLAST and generating a phylogenetic tree using MEGA version 6 software, employing the maximum likelihood method with 1,000 bootstrap replications (11). To evaluate the nucleotide sequence similarity between the two species, the targeted sequences were aligned and compared across various provinces using the EMBOSS Needle and Clustal Omega software. This approach facilitates the identification of regions of high similarity and dissimilarity between the two species, thereby providing valuable insights into their evolutionary relationships.

To identify the presence or absence of specific nucleotide mutations (T917I, L920F, I927F, L928A, R929V, L930M, L932M) within the kdr located in domain II (S5-S6) of the VGSC gene in *L. stenopsis*, we aligned the VGSC gene nucleotide sequences from our samples and analogous sequences from GenBank using the BLAST program. Subsequently, we employed the Expasy-Translate Tool software to convert both the nucleotide sequences of our samples and those of homologous samples from GenBank into their corresponding amino acid sequences. These amino acid sequences were then aligned using CLUSTALW and tool pairwise alignment (emboss-needle) software, allowing us to investigate alterations in the amino acid composition.

## Results

### Morphological and molecular identification

**Figure 1:**
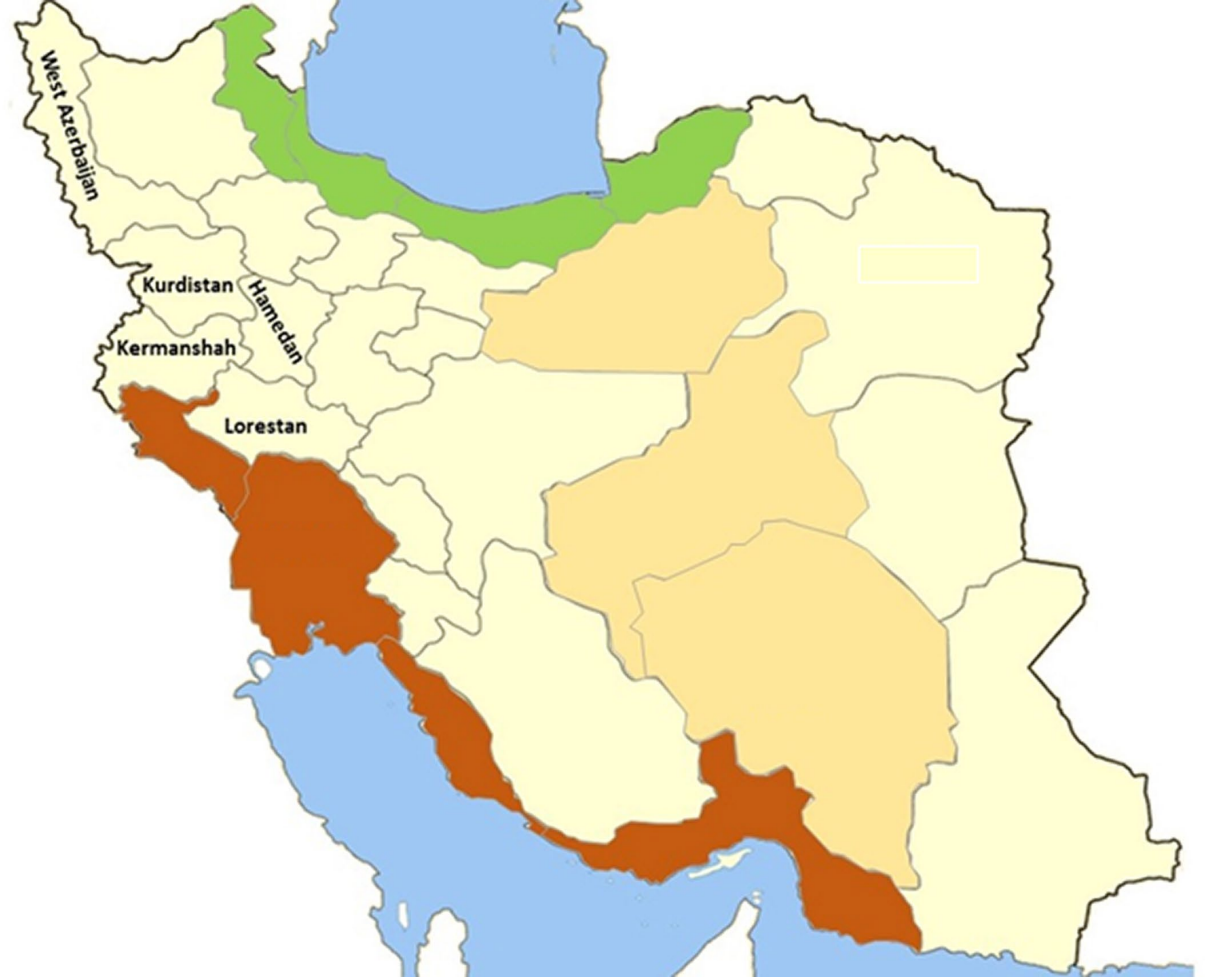
Geographical map of locations where *Linognathus stenopsis* samples were collected in the present study.

In this study morphological identification and molecular assays employed to accurately classify 555 lice of *L. stenopsis* from 1,572 lice collected from 420 goats in 20 farms five provinces (Figure 1). The collected lice were initially identified as *L. stenopsis* by using standardized diagnostic keys under a stereomicroscope. Species identification was achieved by meticulously examining the morphological characteristics of the lice, as described by Wall and Shearer (12, 13). *L. stenopsis* is morphologically characterized by a pale-yellow body color and gray abdomen. It exhibited no eyes or eyespots and possessed five segmented tentacles. The head was narrowly conical, slightly rounded at the anterior margin, and progressively widened towards the thorax. The thorax was relatively short and square with a slight protuberance on the abdomen. The respiratory apertures are conspicuously visible, and the posterior tibia is more developed than the tibiae of the other legs (14).

### Molecular detection of specific allele

**Figure 2:**
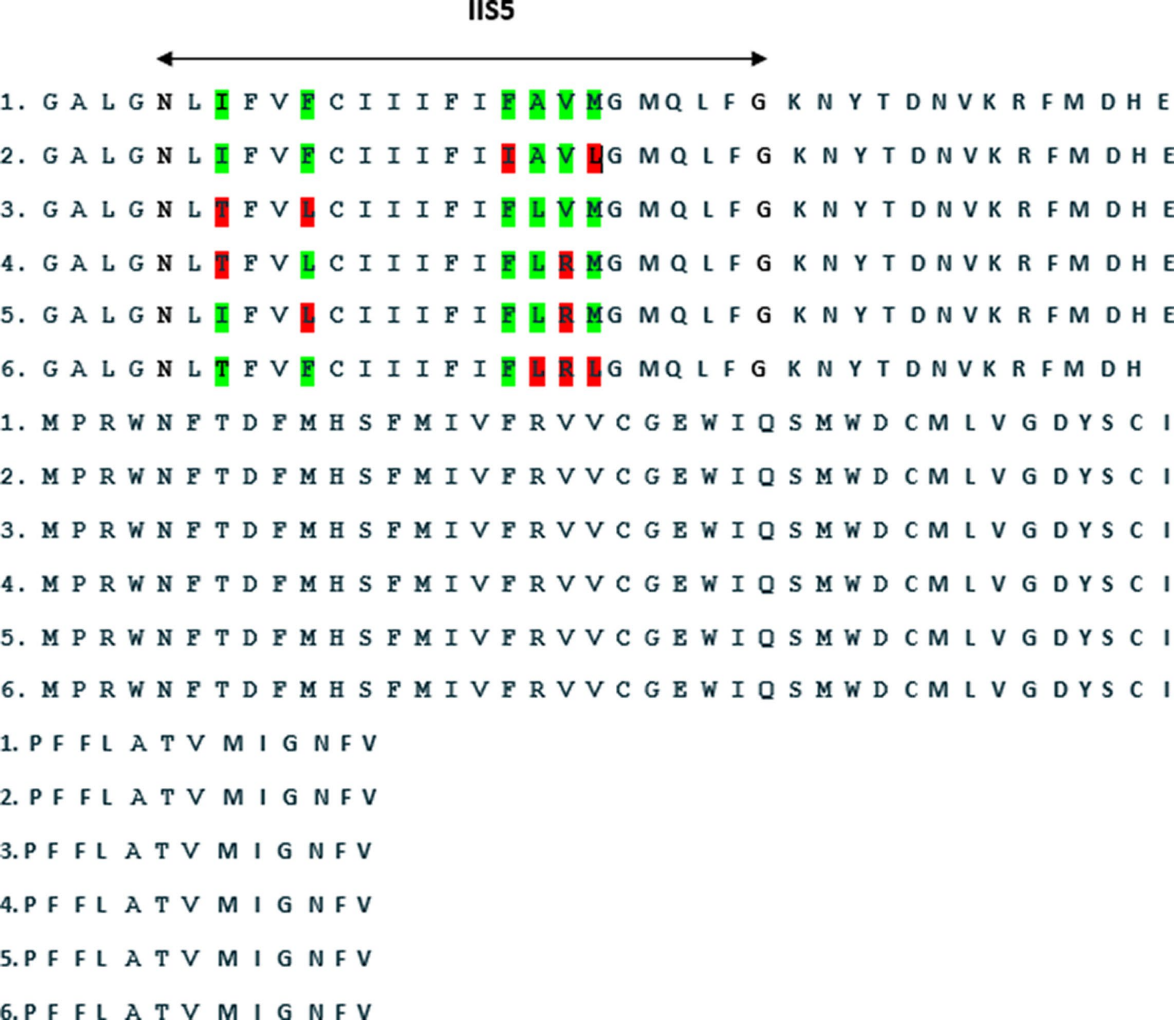
An amino acid sequence alignment of the voltage-gated sodium channel (VGSC) in domain II and the S5-S6 transmembrane region of the VGSC in *L. stenopsis.*

A comprehensive mutational analysis of the VGSC gene in 11 *L. stenopsis* isolates from five provinces revealed six amino acid substitutions (Figure 2). Threonine-to-isoleucine (T917I) was observed in two isolates from Kurdistan and one isolate each from Kermanshah and Hamedan. Leucine-to-phenylalanine (L920F) was detected in one isolate each of West Azerbaijan and Kurdistan. Isoleucine-to-phenylalanine (I927F) substitutions were observed in two isolates from Lorestan. Phenylalanine-to-alanine (F928A) mutations were found in one isolate each from Hamedan and Lorestan. Valine-to-arginine (V929R) alterations were detected in two isolates each from Hamedan, Kurdistan, Kermanshah, and West Azerbaijan. Arginine-to-leucine (R930L) substitutions were observed in two isolates each from Lorestan and Hamedan. However, two isolates from Kermanshah and one from West Azerbaijan exhibited no amino acid substitutions. Figure 2 depicts the comparative alignment of amino acid sequences from the VGSC domain II and S5-S6 transmembrane region in *L. stenopsis* louse specimens.

## Discussion

Classical identification of louse species relies on morphological characteristics, but the variability and similarity of these features often hinder accurate species and subspecies identification. To address this challenge, researchers have employed molecular markers including mitochondrial and nuclear ribosomal genes to offer more precise species differentiation. Mitochondrial genes and Cox1 are suitable genetic markers that are fundamental for successful evolutionary studies (15). And have revolutionized louse species identification approaches by overcoming the limitations of morphological methods, providing a more precise and robust means of distinguishing louse species and subspecies, particularly in cases of morphological similarity. Furthermore, the high mutation rate, maternal inheritance, and conserved nature of the mitochondrial genome make it a valuable tool for evolutionary studies and species identification. Cox1, in particular, has emerged as a highly reliable marker for louse species differentiation and has wide applications in DNA barcoding (16). Molecular techniques have emerged as powerful tools in entomological research, providing a more precise and reliable approach to address a wide range of research questions, including taxonomy, systematics, population genetics, pesticide resistance, and parasitic contamination detection. These techniques have revolutionized our understanding of insect diversity, ecology, and evolution, overcoming the limitations associated with traditional morphological methods (17). The consistency between the findings of the present study and previous studies using morphological analyses further reinforces the reliability of these methods for identifying *L. stenopsis* in goats. However, Tavasoli et al. relied solely on morphological analysis to identify *Bovicola caprae* in goats (18). The absence of molecular analyses in this study limited the definitive identification of the louse species. The utilization of both morphological and molecular techniques in the present study has provided more robust evidence for the presence of *L. stenopsis* in goats from northwest Iran. The *L. stenopsis* sequence identified in this study was deposited in GenBank for the first time to further establish the presence and genetic diversity of this louse species in Iran.

The development of resistance to pyrethroids, has become a major challenge for pest control. One of the primary mechanisms of resistance is kdr, a heritable trait associated with neural insensitivity to pyrethroids that was first discovered in houseflies (19). Over the past two decades, more than 50 mutations or combinations of mutations in the VGSC gene have been identified as contributors to kdr in various arthropods and disease vectors (6, 20). Among these, three-point mutations at positions M815I, T917I, and L920F in the VGSC gene of head lice are particularly prevalent worldwide and are responsible for kdr in pyrethroid-resistant head lice. The emergence of kdr in head lice was first reported in Massachusetts and Florida, USA, where the bioassay results indicated resistance to a class of pyrethroids. Subsequent genetic analysis revealed the presence of three point mutations, M815I, T917I, and L920F, in the VGSC gene of pyrethroid-resistant head lice (21, 22). These mutations are functionally characterized as kdr-type mutations, indicating their ability to confer resistance to pyrethroids (23). Studies have shown that each mutation contributes to reduced pyrethroid sensitivity. However, the T929I mutation, which has been functionally confirmed to be kdr-like in the diamondback butterfly (24), is primarily responsible for resistance in the head lice (23). The frequency of the kdr-like allele in head lice varies between countries, ranging from 44% in Wales and England to 96% in Florida (25). This variation highlights the widespread effect of kdr on the effectiveness of pyrethroid insecticides in combating head lice infestations. In another study, Bass *et al* investigated kdr-like mutations in *Ctenocephalides* felis, a cat flea, and identified the T929V mutation along with the L1014F mutation in the VGSC gene, which contributed to pyrethroid resistance in these fleas (26). Similarly, Ghavami et al. reported the presence of the T929V mutation along with the L1014F mutation in the VGSC gene of *Pollex irritans*, a human flea, which confers pyrethroid resistance (27). These findings further underscore the pervasiveness of kdr mutations in the flea species.

The T917I and L920F substitutions were observed in the *L. stenopsis* sequences examined in this study. An association between T917I and L920F mutations in the VGSC gene and resistance to permethrin has been established in head lice (21, 22). Hodgdon et al reported the presence of only the T917I mutation in some head lice collected from Egypt (28). The present study expands on these findings by identifying additional mutations in the VGSC gene of *L. stenopsis*. These mutations included L928A, R929V, L930M, and I927F. Interestingly, Firozian et al. recently reported the presence of new mutations in the VGSC genes of head and human body lice, including M930L, V929R, and A928L, in addition to the well-established T917I and L920F mutations (29). However, the functional significance of these mutations in pyrethroid resistance remains unclear. In the present study, the M930L, V929R, and A928L mutations were also identified in the VGSC gene sequence of *L. stenopsis* lice. Additionally, the novel I927F mutation was observed in *L. stenopsis* but was not observed in the study by Firooziyan et al. (29).

The emergence of these VGSC gene mutations in *L. stenopsis*, particularly in regions where pyrethroids are extensively used, suggests the potential development of resistance to these insecticides in this species. This raises concerns regarding the sustainability of pyrethroids in controlling *L. stenopsis* infestations. Additional research is necessary to clarify the functional implications of these mutations and devise efficient approaches for addressing lice infestations in goats. Despite substantial advancements in our understanding of kdr mutations, several critical questions remain unresolved. Which physiological and evolutionary factors restrict the emergence and frequency of the kdr alleles in the field? Although it is evident that kdr mutations at the predicted pyrethroid receptor site confer resistance, the mechanism by which mutations outside this site contribute to resistance remains elusive. Could there be alternative pyrethroid receptor sites in sodium channels? Could the altered channel gating (activation, deactivation, and/or inactivation) caused by kdr mutations counteract the action of pyrethroids and contribute to resistance? Addressing these lingering questions is paramount for the development of effective strategies to combat pyrethroid resistance (6). Control of louse infestations is essential whenever an animal exhibits excessive scratching and rubbing behavior. Louse control with a single insecticide application is challenging because it fails to eliminate louse eggs. A second application is necessary 2 weeks following the initial treatment to target newly hatched louse larvae (30). This two-step approach ensures comprehensive louse elimination and alleviates animal discomfort caused by infestation.

The study’s sample size of 555 lice collected from goats in five provinces of Iran was relatively low, which could limit the generalizability of the findings to larger goat populations in Iran and other parts of the world. Moreover, this study only examined a single region of the VGSC gene, which harbors multiple mutations that can contribute to pyrethroid resistance. This suggests that there may have been other mutations in the VGSC gene of *L. stenopsis* lice that were not detected in this study. Additionally, this study did not assess the functional significance of the identified mutations, which means that it is not known whether these mutations confer resistance to pyrethroids. Further studies are needed to investigate the functional effects of these mutations. Due to the limitations of this study, we could not compare the prevalence of mutations in the VGSC gene of *L. stenopsis* from different regions of Iran. This information would have helped us to understand how the distribution of mutations may vary depending on the use of pyrethroids in different regions. Despite these limitations, this study provides important information regarding the prevalence of pyrethroid-resistance mutations in *L. stenopsis* lice in Iran.

## Conclusion

The findings of this study, which identified the T917I, L920F, L928A, R929V, L930M, and I927F mutations in the VGSC gene of *L. stenopsis* lice from Iran, underscore the potential development of pyrethroid resistance in this louse species. These findings reinforce the need for integrated pest management approaches that combine multiple control methods, including selective insecticides, to effectively manage *L. stenopsis* infestations and to maintain the efficacy of pyrethroids for pest control.

## Data availability statement

The original contributions presented in the study are publicly available. This data can be found at: https://www.ncbi.nlm.nih.gov/; ON455013-ON455022.

## Ethics statement

The animal study was reviewed and approved by the Ethics Committee of Urmia University of Medical Sciences. Written informed consent was obtained from the owners for the participation of their animals in this study.

## Author contributions

KS-G: Writing – review & editing, Writing – original draft. MT: Writing – review & editing, Writing – original draft. KM: Writing – review & editing, Writing – original draft.
